# P08-15 More active parents, more active children: Association between mode of commuting and physical activity

**DOI:** 10.1093/eurpub/ckac095.128

**Published:** 2022-08-29

**Authors:** Fernando Rodríguez-Rodríguez, Francisco Javier Huertas-Delgado, Javier Sevil-Serrano, Yaira Barranco-Ruiz, María Jesús Aranda-Balboa, Palma Chillón

**Affiliations:** 1 IRyS Group, School of Physical Education, Pontificia Universidad Católica de Valparaíso, Viña del Mar, Chile; 2 Physical Activity for Health Promotion, Research Group, La Inmaculada Teacher Training Centre., University of Granada, Granada, Spain; 3 PROFITH ‘PROmoting FITness and Health through physical activity' research group, Department of Physical Education and Sport, Faculty of Sport Sciences, University of Granada, Granada, Spain

**Keywords:** School, Schoolchildren, Active transport, Girls, Boys

## Abstract

**Background:**

A substantial body of research have shown that parents' behaviours are closely linked to children's behaviours. Although some studies have reported positive association between parents' and youths' physical activity, especially in same sex (father-son; mother-daughter), further studies are required to strengthen these preliminary findings. The main objective of this study was to examine the relationship between fathers' and mothers' physical activity and mode of commuting and sons' and daughters' physical activity and mode of commuting.

**Methods:**

This cross-sectional study included 1,372 participants, 686 parents (43.4±6.5 years; mothers: 52.8%) and 439 children (age: 9.7±1.7 years; girls: 65.1%) and 246 adolescents (14±1.7 years; girls: 68.3%). Each participant completed a self-report questionnaire on physical activity (Parents: IPAQ; Children: YAP) and commuting patterns (PACO: Pedalea y Anda al Cole). The parents completed and signed an informed consent on the characteristics of the study, which was approved by the corresponding ethics committee. Descriptive statistics (mean, standard deviation, and frequency distribution) and chi-square test (p > 0.05) were used to examine this association. Odds ratio (OR) and confidence interval 95% (CI) were used to determine the degree of association.

**Results:**

Most of the sample did not meet Physical activity recommendations, particularly adolescents and parents (children: 46.4%; adolescents: 6.5%; and parents: 7.2%). Less than one third of the sample commute to school/work actively (children: 31.6%; adolescents: 31.0%; and parents: 31.6%). Only the fathers' physical activity was positively associated with their female childreńs physical activity (OR = 2,477, 95%CI: 1,144-5,362). A positive association between fathers' active commuting to work and their children' commuting to school was only found in girls (Girls OR = 2,890, 95%CI: 1,336-6,251), but this relationship was not found in boys' children and adolescents. And another association was found between motherśactive commuting to work and their childreńs commuting (Girls OR = 3,242, 95%CI: 1,343-7,828; Boys OR = 6,381, 95%CI: 2,530-16,091), but not with adolescents.

**Conclusions:**

Parent's physical activity and active commuting to work are closely linked to their children's physical activity and active commuting to school, especially in girls. This study emphasizes the importance to involve parents in school-based interventions to create a positive ripple effect in Physical activity-related behaviours.

